# A longitudinal, randomized experimental pilot study to investigate the effects of airborne ultrasound on human mental health, cognition, and brain structure

**DOI:** 10.1038/s41598-021-83527-z

**Published:** 2021-03-12

**Authors:** L. Ascone, C. Kling, J. Wieczorek, C. Koch, S. Kühn

**Affiliations:** 1 Department of Psychiatry and Psychotherapy, Neuronal Plasticity Working Group, University Medical Center Hamburg-Eppendorf, Martinistr. 52, 20246 Hamburg, Germany; 2 Max Planck Institute for Human Development, Lise Meitner Group for Environmental Neuroscience, Lentzeallee 94, 14195 Berlin, Germany; 3 Physikalisch-Technische Bundesanstalt Braunschweig, Bundesallee 100, 38116 Braunschweig, Germany

**Keywords:** Neuroscience, Psychology, Risk factors

## Abstract

Ultrasound-(US) emitting sources are highly present in modern human environments (e.g., movement sensors, electric transformers). US affecting humans or even posing a health hazard remains understudied. Hence, ultrasonic (22.4 kHz) vs. sham devices were installed in participants’ bedrooms, and active for 28 nights. Somatic and psychiatric symptoms, sound-sensitivity, sleep quality, executive function, and structural MRI were assessed pre-post. Somatization (possible nocebo) and phasic alertness increased significantly in sham, accuracy in a flexibility task decreased significantly in the verum condition (indicating hastier responses). Effects were not sustained after *p*-level adjustment. Exploratory voxel-based morphometry (VBM) revealed regional grey matter (rGMV) but no regional white matter volume changes in verum (relative to placebo). rGMV increased in bilateral cerebellum VIIb/Crus II and anterior cingulate (BA24). There were rGMV decreases in two bilateral frontal clusters: in the middle frontal gyri/opercular part of inferior frontal gyrus (BA46, 44), and the superior frontal gyri (BA4 ,6, 8). No brain-behavior-links were identified. Given the overall pattern of results, it is suggested that ultrasound may particularly induce regional gray matter decline in frontal areas, however with yet unclear behavioral consequences. Given the localization of clusters, candidate behavioral variables for follow-up investigation are complex motor control/coordination, stress regulation, speech processing, and inhibition tasks.

**Trial registration**: The trial was registered at NIH www.clinicaltrials.gov, trial identifier: NCT03459183, trial name: SonicBrain01, full trial protocol available here: https://clinicaltrials.gov/ct2/show/NCT03459183.

## Introduction

It is now known that we are—mostly without our conscious awareness—exposed to ultrasound in our modern, technologized environments each and every day^1^. As a rule of thumb, the term ultrasound comprises frequencies above the upper human hearing threshold (∼20 kHz)—however depending upon the sound pressure level (SPL), even higher frequencies are audible (or rather otherwise ‘perceivable’). Hearing thresholds could be determined at frequencies above 20 kHz^1–3^ demonstrating an auditory perception at least in a subset of test persons. The experiments showed the highly individual properties of this perception process. A recent study identified a multitude of ultrasonic devices in public areas, some of them emitting ultrasound with remarkably high SPLs (e.g., pest deterrents emitted ultrasound of up to 100 dB SPL at frequencies of about 20 kHz). Other identified sources in that study included a hand dryer, a door sensor, public voice alarm systems, or a cathode ray tube television^2^.

Adverse effects (e.g., fatigue, vertigo, heart rhythm disturbances, lack of concentration, anxiety, irritability, memory and learning problems) have been occasionally documented for ultrasonic noise (which contains a mixed spectrum of both audible, high-frequency noise and inaudible ultrasonic components), mainly in earlier studies with industrial workers, and field studies on individuals exposed to ultrasound^1,4^. It has been found that noise that contains ultrasonic components, for instance in a study using an ultrasonic washer (including a frequency spectrum from 1.6 kHz to 80 kHz and A-weighted SPLs of 72 dB, 80 dB and 96 dB), is rated as particularly unpleasant and annoying, even at the lowest levels^5^. In another study, using an animal repellent system as a source, no indications of significant adverse effects were identified^6^. The system was audible to some of the test persons, and for those it was disturbing. For inaudible airborne ultrasound, there is a general lack of well-controlled studies in order to estimate potential health hazards and investigate any effects driven by psychological variables, such as expectation or attitude (e.g., conviction about the dangerousness of ultrasonic frequencies). Small nocebo effects (on ear pain, dizziness, and tinnitus) have recently been reported in double-blind trial, exposing individuals to a 20 kHz inaudible tone vs. sham for 15 minutes. In this trial, ultrasound exposure per se did not evoke any increases in self-reported adverse symptoms, but apparently the expectation to be exposed drove the effects. This pattern of results was replicated in an individual with high self-reported ultrasound sensitivity. The study nevertheless concluded that long-term effects may differ from this result^7^. Controversially, there is also evidence for increased pleasantness through ultrasound. One study reported a musical preference for a piece with (vs. without) ultrasonic components (above 22 kHz), and the occipital alpha-EEG signal was enhanced alongside with increases of regional cerebral blood flow in the left thalamus, as measured using PET^8^. In general, however, the investigation of brain effects of ultrasound is in its infancy. When it comes to ‘pure’ ultrasound, an fMRI study found no evidence of auditory cortex activation for airborne ultrasound below the hearing threshold^3^, as already documented in an earlier MEG (magnetencephalography) that did not find auditory evoked magnetic field changes in response to frequencies at or above 20 kHz^9^.

In sum, the evidence of effects ultrasound on the human brain or human health can be said to be under-researched and ambiguous. Long-term exposure studies are lacking, and methodologically rigorous randomized-controlled trials are urgently needed. It is well-known that our immediate environment and lifestyle can have a profound impact on the brain’s morphology, and ultrasound is now a common stimulus in our immediate environment. Hence, studying this can be said to be not only relevant, but necessary. The few existing trials used a laboratory setting with only brief stimulations, which does not allow for assessing more sustainable effects. For those reasons, the present study was set out as the first ever conducted randomized long-term exposure trial (1 month) of humans to airborne ultrasound vs. a sham condition, addressing the issue of significant effects on human (mental) health, cognition, and brain structure. Effects of inaudible US on human mental health (i.e., psychiatric symptoms in general, anxiety, depression, stress), somatic symptoms (i.e., sleep disturbances, somatic symptoms), cognition/attention (i.e., alertness, vigilance [sustained attention], cognitive flexibility, divided attention, attention shifting, inhibition), and brain structure have never been studied comprehensively before, which is the intention of the present study.

## Methods

### Recruitment and in- and exclusion criteria

The trial was pre-registered in the National Institute of Health trial registry (a full trial protocol is available here: https://clinicaltrials.gov/ct2/show/NCT03459183, trial identifier: NCT03459183; ID: SonicBrain01; registration date: 08/03/2018). In all procedures, we adhered to the declaration of Helsinki, and the study was approved by a local ethics board prior to study onset (Ethik-Kommission der Ärztekammer Hamburg; approval number: PV5570). Active recruitment and data collection took place between May 31, 2018 until December 15, 2019. We obtained informed consent from all study participants who were enrolled in the study. The study comprised a 2 (ultrasound verum vs. placebo [sham]) × 2 (pre-post 1 month of sound exposure) repeated-measures randomized-controlled, single-blind (participants unaware of group assignment) design. Participants were assigned to the conditions based on list-wise randomization, with an allocation ratio of 50:50. The randomization list included a computed-generated random sequence which was implemented by the first author (L.A.), and restrained to 25 slots per condition (considering potential drop out). Included participants were sequentially assigned to the next available list position and unaware of their assignment until the end of the trial. The experimenter was aware of the group assignment. Several advertisements in local newspapers were run, and flyers systematically spread across the city of Hamburg, searching for healthy test persons. Interested individuals who contacted the study team first received exhaustive study information and a link for an online screening to check in- and exclusion criteria. The screening took about 45 min, and included socio-demographic assessments, including sex, age (required to be between 18 and 45 years), education, partnership status, children, regular medication intake, and variables addressing housing conditions (incl. size of the bedroom, number of windows and doors in the bedroom, city district, and closeness to main roads). Children sleeping in the same bedroom was an exclusion criterion for safety reasons. In addition, we advised pet owners to keep their animals outside the room for the time of the exposure. Main exclusion criteria were counter-indications for magnetic resonance imaging (MRI) (i.e., cochlear implants, non-removable metal on/in the body, or tinnitus), chronic inflammatory, autoimmune, or other severe illnesses (e.g., cancer), as well as central-nervous system diseases. Similarly, indicating any anomalies concerning hearing (e.g., deafness, past ear surgery, chronic inflammation of the ear canal, chronic sinusitis, anatomic anomalies) lead to exclusion. Central nervous medication intake or participation in a medical trial also led to exclusion. Further health-relevant variables assessed were smoking and alcohol consumption. Mental illness was assessed using standardized screening tools: the Mini International Neuropsychiatry Interview (MINI)^10^ for axis I disorders, and the Structured Clinical Interview for DSM-IV-II (SCID-II)^11^ for axis II (personality) disorders. Only the screening questions of the respective interviews were included in the online survey. Any positive screening was followed up on in a subsequent telephone interview, which, depending upon the amount of positively endorsed clinical screening questions, could take between 20 and 60 min. Telephone contacts also included providing further information on the study and answering the participants’ questions. Medical or psychological student research assistants who were trained and supervised by a postdoc level clinical psychologist conducted all telephone screenings. Suspicion of a potential mental disorder led to exclusion from the study.

A minimum sample size of N ≈ 40 was determined based on previous experience of the principal investigator (S. K.) who is an expert in conducting research of neuroplasticity induced by environmental changes that were observed in comparable samples after experimental interventions between 4 and 8 weeks. This constituted a minimum compromise based on available time and resources.

### Study procedure

If all in- and exclusion criteria were fulfilled, appointments for the pre-test, on-site sound source installation, and post-test were made. Both assessments before and after the exposure took place at the neuroplasticity research laboratory unit at University Medical Center Hamburg-Eppendorf and were divided into two blocks. A first block included intermixed self-reports (self-reports of somatic and mental illness symptoms, sleep quality self-reports, personality tests) and cognitive tasks (e.g., alertness, inhibition, task switching, working memory, sustained attention) (2–2.5 h in total). A second block included a MRT session (1–1.5 h), where also a spatial n-back task was performed in the scanner. Closely after the pre-test assessment (one day to maximally one week after initial assessment), participants were randomly assigned to one of the ultrasound-verum vs. -sham groups, and the on-site sound source installation took place. For the entire process, we followed a standardization of procedure protocol (see Supplementary Appendix I for details). The ultrasound sources were commercially available devices, which were modified for the purpose of the experiment, and which emitted a frequency of about 24.2 kHz. Due to the lack of a reliable ultrasound level meter, the SPL was not explicitly measured in-situ, but the sources were adjusted in preceding laboratory tests to a maximum emitted sound pressure level. The sources were configured so that they emitted sound steadily for eight hours during the participant’s self-reported, habitual sleep time (refer to Supplementary Appendix I for details). They were installed close to the bed on loudspeaker-stands that were adjusted to match the height of the head-position during sleep. The sources were chosen for general and practical reasons. Animal repellent systems are quite common and represent a typical ultrasound source in public spaces. They are easy to handle and could be adapted to the required experimental settings with simple modifications of the circuitry. The sham sources looked and operated identical to the verum sources but did not emit any sound. For a detailed description of the design, technical details, and initial calibration of the US sources and for descriptive data on the on-site constellation details and exposure levels please refer to Supplementary Appendix I. After 28 days of exposure, the post-test, with the same measures, taken in the same order as at pre-test, took place.

### Measures

#### Self-reports

All self-reports were assessed always in the same order for all participants at all assessment points (baseline, post-test) and filled out by the participants on a computer.

The Brief Symptom Inventory (BSI)^12^ was used to measure global severity of psychiatric symptoms. It contains 53 items, asking for how strongly respondents were affected (0 = *not at all*, 4 = *extremely*) by a range of different problems, which can be categorized into nine symptom group subscales. We separately analyzed the somatization, depression, and anxiety subscales. The BSI has been shown to have sufficient to excellent reliability with Cronbach’s αs of 0.90 for the global severity index, 0.63 for somatic symptoms, 0.62 for anxiety, and 0.72 for depression. Participants were instructed to rate symptoms for the past two weeks.

The Perceived Stress Scale (PSS) measures the perceived stressfulness of daily life situations. Fourteen items address how often the respondents felt stressed (vs. in control of things) on a 5-point frequency scale ranging from 0 (= *never*) to 4 (= *very often*). Reliabilities (Cronbach’s α) have been reported as good (0.84–0.86)^13^. Again, participants answered the questions referring to the last two weeks.

In order to assess daytime sleepiness and fatigue, the Epworth Sleepiness Scale (ESS) was used^14^. It asks the participant to rate the perceived likelihood of dozing in eight typical daytime activities (e.g., sitting quietly after a lunch without alcohol; 0 = *would never doze*, 1 = *slight chance of dozing*, 2 = *moderate chance of dozing*, 3 = *high chance of dozing*). The scale refers to daily life in recent time. Good reliability (Cronbach’s α of 0.88) has been reported^15^.

Overall quality or disturbances of sleep was assessed using the Pittsburgh Sleep Quality Inventory (PSQI)^16^. Seven components, based on the participants’ replies to 19 questions, are evaluated: subjective sleep quality, sleep latency, sleep duration, habitual sleep efficiency, sleep disturbances, use of sleeping medication, and daytime dysfunction. The scores can range between 0 and 21, as each component is rated from 0 to 3, with lower ratings indicating poorer sleep quality. The components are usually integrated into a single global sleep quality score. The PSQI has been shown to sensitively differentiate between good and poor sleepers^16^. Reports in our study referred to the last 2 weeks.

Particularly neuroticism and introversion have been shown to be related to higher sensitivity, perceived loudness, and annoyance induced by high-frequency noise (e.g.,^17^). Hence, in order to make sure that there were no differences in this variable between the groups at baseline, a 30-item version of the five factor personality inventory (NEO-FFI-3)^18^ was assessed. This questionnaire assesses *neuroticism* (characterized by ‘moodiness’ and frequent experience of aversive as emotions), *extraversion* (enjoying human interactions, enthusiasm and zest, talkativeness, assertiveness, and gregariousness), *conscientiousness* (orderliness, self-discipline, dutifulness, competence, achievement striving, and deliberation), *openness* (intellectual curiosity, aesthetic sensitivity, attentiveness to feelings, preference for variety) and *agreeableness* (warmth, kindness and empathy). All items are rated on a 5-point Likert-scale, ranging from 0 = *strongly disagree* to 4 = *strongly agree*. Both factorial validity and good internal consistencies (Cronbach’s α) have been reported for all subscales, ranging between 0.78 and 0.86^18^.

In addition, sound sensitivity was assessed. Normal (hearing sound) sensitivity was quantified using the Noise Sensitivity Questionnaire which has been reported to have excellent reliability (.90),^19^. The questionnaire comprises 35 items rated on a 4-point Likert scale (*strongly agree* = 3, *slightly agree* = 2, *slightly disagree* = 1, and *strongly disagree* = 0). Sensitivity to high frequency sound in particular was assessed using the SISUS-Q (sensitivity to infra- and ultrasound questionnaire) which is a brief and economic scale consisting of four items, rated on an 11-point Likert (0 = totally disagree, 10 = totally agree) that assess high-frequency-sensitivity with good reliability (Cronbach’s alpha = .82),^20^.

#### Cognition

We used the computer-based Tests of Attentional Performance (TAP)^21^ to assess a set of cognitive performance indicators in several domains, namely alertness, sustained attention, flexibility, divided attention, incompatibility (Simon task), covert shift of attention, and inhibition (GoNogo). The choice to investigate executive functioning was made to reduce the complexity of reported adverse cognitive effects (e.g., reduced concentration, impaired work performance, mnestic and learning problems) and break it down to underlying basic functions. For each test, different parameters are of relevance (see Supplementary Appendix II).

#### MRI scanning parameters

Brain scans were performed with a 3 T Siemens Magnetom Prisma (Siemens Medical Systems, Erlangen, Germany) using a 64-channel head coil. A 3D MPRAGE was run with 256 slices per slab, FOV = 240 mm, TR = 2500 ms, TE = 2.12 ms, TI = 1100 ms, voxel size = 0.8 mm × 0.8 mm × 0.9 mm.

### Statistical analyses

#### Voxel-based morphometry

We performed our pre-processing and whole brain analyses using the toolboxes SPM12 (v7487), (https://www.fil.ion.ucl.ac.uk/spm/software/spm12) and CAT12 (Structural Brain Mapping Group, University of Jena—exact version: CAT12.6-rc1 [r1429] from 2019-02-08), (http://www.neuro.uni-jena.de/cat/index.html). We run the toolboxes with Matlab R2017a (MathWorks Inc., Natick, MA). Pre-processing steps were conducted following the default CAT12 segmentation routine for longitudinal data (http://dbm.neuro.uni-jena.de/cat12/CAT12-Manual.pdf) including registering the segmented images to the MNI space using the high-dimensional Dartel approach^22^.

#### Behavioral data analysis

A series of classical test theory repeated-measures ANOVAs were carried out in SPSS 25 (IBM Corp. 2017) for all variables of interest. Post-hoc exploratory paired t-tests were applied to identify within group changes underlying the interaction. To adjust for multiple testing, we used Bonferroni correction. Effect size *η*^*2*^_*partial*_ was interpreted as *η*^*2*^_*partial*_ > 0.01 small, > 0.06 medium, > 0.14 large effect.

#### Structural brain data analysis

We performed a whole-brain voxel-based morphometric (VBM) analysis with no prior assumptions concerning affected regions of interest (ROIs), as no pre-assumptions could be made due to the lack of research on structural brain effects of long-term US exposure. The analyses were run with the preprocessed, segmented grey matter images using SPM12, examining both global increases and decreases in regional grey matter volumes (rGMV) in the US verum condition, while controlling for, and assuming stability (no change) in the US placebo condition. The following contrasts were computed: with verum [pre, post], placebo [pre, post] relative increase in US verum vs. placebo; contrast: − 1 3 − 1 − 1; relative decrease in US verum vs. placebo; contrast: 1 − 3 1 1). The same analytical approach was repeated to identify any regional white matter volume changes in verum relative to placebo.

In addition, we computed the contrast 0 0 − 1 1 (increases from pre-to-post within the sham condition) and 0 0 1 − 1 (decreases from pre-to-post within the sham condition) to investigate any changes over time in the non-exposed group. Results for the latter two contrasts can be found in detail in the supplementary document (Supplementary Appendix III) but will also be briefly reported in this paper. A flexible factorial design was chosen, establishing a model with group and time factor and their interaction. An absolute threshold masking with a value of 0.01 was set. The resulting maps were thresholded with *p* < 0.001. The statistical cluster extent threshold was applied to correct for multiple comparisons. The latter was combined with a non-isotropic smoothness correction based on permutation as proposed by Hayasaka and Nichols^23^ (as implemented in the CAT12 toolbox).

#### Association of structural with behavioral changes

In a last step, we extracted mean rGMV volumetric data from any identified significant clusters from the VBM analyses using the REX (https://www.nitrc.org/projects/rex) toolbox [release alpha0.5; Neuro Imaging Tools and Resources Collaboratory]. The identified significant clusters (spmT-extent-thresholded-cluster images) from the VBM were used as masks to extract the volumetric information within each of the ROIs, separately for baseline and post-exposure assessments. Afterwards we correlated the changes in volumetric rGMV data for each identified ROI with changes in behavioral data of variables that exhibited a significant change within verum. For variables differing from normality (skew and/ or kurtosis > 2 or < − 2), and/or variables measured at a ranked, rather than interval level (e.g., number of errors), non-parametric correlations were computed (Spearman), as these have additionally been shown to be more robust in case of outliers^24^. For the correlations, we used Cohen’s^25^ rule of thumb to determine effect size: *r* ≥ 0.10 = small effect, r ≥ 0.30 medium effect and *r* ≥ 0.50 = large effect.

### Ethics and participant consent

The study was approved by a local ethics consortium prior to study onset. The study adhered to the declaration of Helsinki. All participants consented to participate in the study.

## Results

### Sample

In total, 34 participants fully took part in the study including both behavioral and neuroimaging data, hence 34 pre-post datasets were available for structural brain or brain-behavior correlation analyses (*n*_verum_ = 20, *n*_placebo_ = 14). One participant (ultrasound verum) even after encouragement by the study team refused to again go into the scanner after an initial strong self-reported aversive (fear) response. This participant was not excluded from the study as a whole, as her partner also took part in the study (i.e., simultaneous sound exposure as a couple). Hence, for behavioral analyses, *n* = 21 cases were available in ultrasound verum. On top of these participants, there were 4 dropouts (all during pretest). Reasons for dropout were claustrophobia in the scanner (3 cases), and mental disorder (1 case) that was revealed during pre-test. Socio-demographic details for each group can be found in Table 1. There were no differences in any of the demographic variables across the groups. There were no adverse events leading to premature study termination. In addition, differences between the groups in personality and sensitivity (high frequency, hearing sound) were tested to exclude confounders. Descriptive data can be found in Supplementary Appendix III. No differences between any of the personality dimensions (openness, neuroticism, conscientiousness, extraversion/ introversion, agreeableness) or sensitivity could be identified (all *p* > 0.30).

**Table 1:** Descriptive sample data and between-group differences for socio-demographic variables.

Variable/descriptives	Ultrasound—verum (*n* = 21)	Ultrasound—placebo (*n* = 14)	Inferential statistics
Age: mean (SD)	27.48 (5.53)	25.57 (5.26)	*t*(33) = 1.02, *p* = 0.316
Sex: percentage male/female (no. male/no. female)	43/57 (9/12)	50/50 (7/7)	*X*^2^ (1, *N* = 35) = 0.17, *p* = 0.678
Years of education	16.83 (3.07)	15.71 (3.58)	*t*(33) = 0.99, *p* = 0.330
Children: percentage yes/no (no. yes/no)	5/95 (1/20)	7/93 (1/13)	*X*^2^(1, *N* = 35) = 0.09, *p* = 0.766
Nationality: percentage German/other (no. German/other)	90/10 (19/2)	86/14 (12/2)	*X*^2^(1, *N* = 35) = 0.19, *p* = 0.664
Regular medication: percentage yes/no (no. yes/no)	5/95 (1/20)	0/100 (0/14)	*X*^2^(1, *N* = 35) = 0.69, *p* = 0.407

### Behavioral results

For descriptive pre-post data please refer to Supplementary Appendix III. Given that 21 hypothesis tests were carried out, the rate of positive results (H_1_) identified based on mere chance equals: 21 × 0.05 = 1.05. The identification of more than one significant result may indicate that one of these findings is genuine. Three significant interactions were discovered (see Table 2): a medium-sized effect for s*omatization* (*η*^*2*^_*partial*_ = 0.133, *p* = 0.037; attributable to significant increases in placebo [paired t-tests within groups]: *t* (13) = 2.38, *p* = 0.034, *d* = 0.63; verum: *t* (19) = 0.53, *p* = 0.603), a large effect for the *phasic arousal index* (*η*^*2*^_*partial*_ = 0.207, *p* = 0.007; significant increase in placebo: *t* (13) = 4.04, *p* = 0.001, *d* = 1.08; verum: *t* (20) = 1.21, *p* = 0.241) and a large effect for the *speed-accuracy trade-off* (*η*^*2*^_*partial*_ = 0.204, *p* = 0.007; significant decrease in verum [shift towards speed strategy]: *t* (20) = 2.15,* p* = 0.044, *d* = 0.47; placebo: *t* (13) = 1.97, *p* = 0.071). Bonferroni-correction sets the significance needed to reject the H_0_ to *p* < 0.0024. None of the identified results remained significant after applying the correction.

**Table 2:** Results (group × time interaction effects) of the repeated measures ANOVAs for all behavioral variables.

Dependent variables	Statistics for the interaction effect
**Sensitivity**	
High frequency sensitivity	*F*(1,33) = 0.18, *p* = 0.673, *η*^2^_p_ = 0.005
Normal sound sensitivity	*F*(1,33) = 0.19, *p* = 0.732, *η*^2^_p_ = 0.004
**Symptoms and sleep**	
BSI total	*F*(1,32) = 2.91, *p* = 0.098, *η*^2^_p_ = 0.083
**BSI somatization**	***F(1,32) = 4.93, p = 0.034, η***^**2**^_**p**_** = 0.133**
BSI depressive symptoms	*F*(1,32) = 0.62, *p* = 0.439, *η*^2^_p_ = 0.019
BSI anxiety symptoms	*F*(1,32) = 2.18, *p* = 0.150, *η*^2^_p_ = 0.064
ESS sleepiness	*F*(1,33) = 1.43, *p* = 0.240, *η*^2^_p_ = 0.042
PSQI—sleep quality (total)	*F*(1,30) = 0.08, *p* = 0.784,* η*^2^_p_ = 0.003
PSS perceived stress	*F*(1,33) = 3.36, *p* = 0.076, *η*^2^_p_ = 0.092
**Alertness**	
Median RTs tonic arousal	*F*(1,33) = 1.72, *p* = 0.199, *η*^2^_p_ = 0.049
Median RTs phasic arousal	*F*(1,33) = 0.71, *p* = 0.405, *η*^2^_p_ = 0.021
**Phasic alertness index**	***F(1,33) = 9.01, p = 0.005, η***^**2**^_**p**_** = 0.214**
Anticipations after warn tone (low impulse control)	*F*(1,33) = 0.51, *p* = 0.480, *η*^2^_p_ = 0.015
**Sustained attention (WM)**	
Omissions (total)	*F*(1,33) = 0.90, *p* = 0.349, *η*^2^_p_ = 0.027
**Flexibility**	
**Speed-accuracy index**	***F(1,33) = 7.28, p = 0.011, η***^**2**^_**p**_** = 0.181**
Total performance index	*F*(1,33) = 1.61, *p* = 0.214, *η*^2^_p_ = 0.046
**Divided attention**	
Omissions (total) *Incompatibility*	*F*(1,33) = 0.33, *p* = 0.569, *η*^2^_p_ = 0.010
Incompatibility effect (visual field × hand)	*F*(1,33) = 0.99, *p* = 0.328, *η*^2^_p_ = 0.029
Errors incompatible	*F*(1,33) = 0.06, *p* = 0.816, *η*^2^_p_ = 0.002
**Covert shift of attention**	
Validity × side (re-orientation of attention)	*F*(1,33) = 0.12, *p* = 0.736, *η*^2^_p_ = 0.003
**GoNoGo (inhibition)**	
Errors (total)	*F*(1,32) = 0.06, *p* = 0.802, *η*^2^_p_ = 0.002

Alpha level = 0.05; Bonferroni-adjusted = 0.05/21 = 0.0024. After application of the corrected *p*-level none of the significant effects (reported here with unadjusted level, highlighted in bold in Table) remain. The likelihood of by chance detecting a significant result is 5%, with 21 tests this equals 1.05 tests that would be detected as significant by pure chance.

### Brain structure results

#### Increases and decreases in ultrasound verum relative to placebo in regional grey matter volume

**Figure 1:**
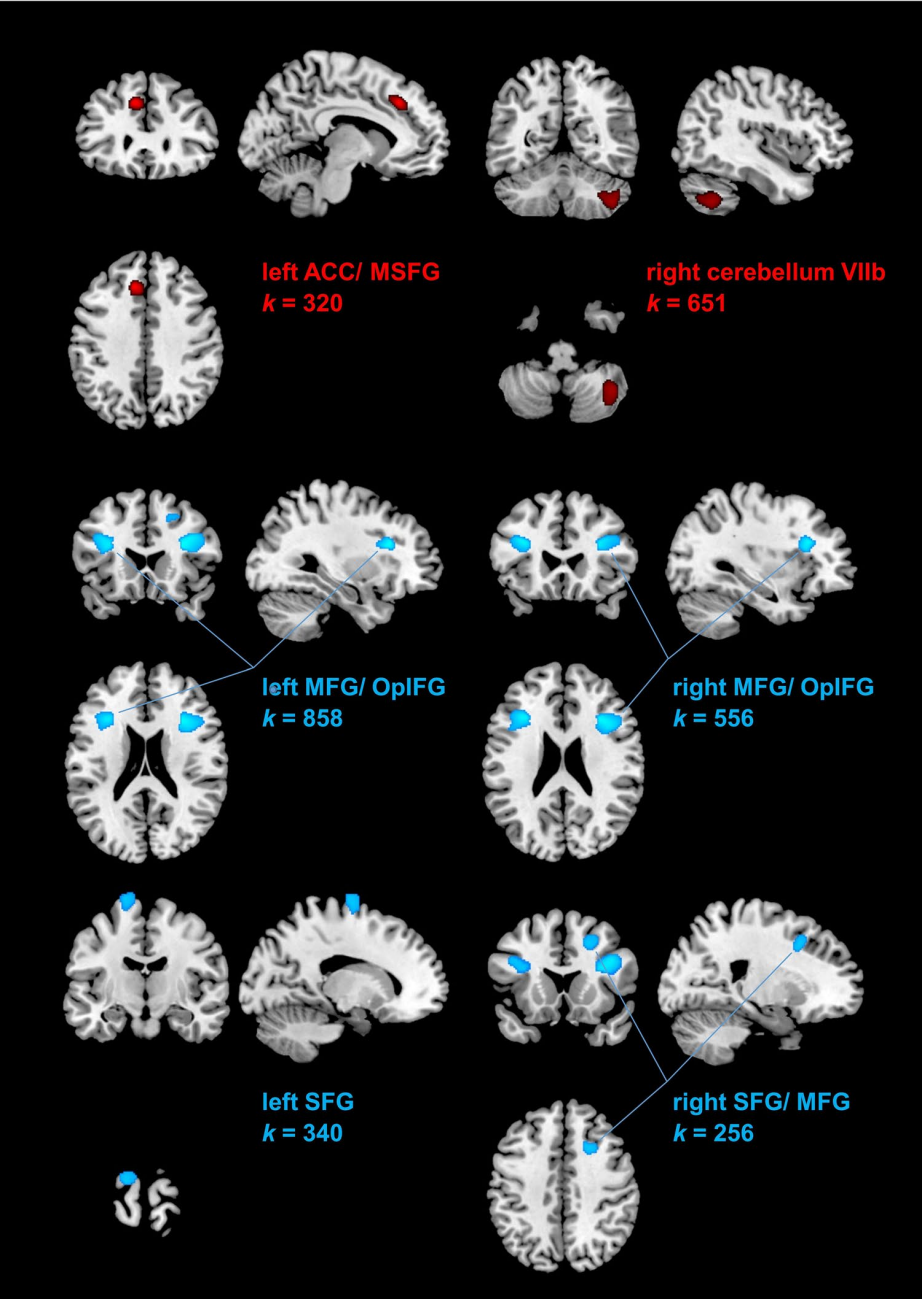
Graphical depiction of identified significant clusters in the VBM analysis of increases in rGMV (in red): in the left anterior cingulate cortex (ACC)/medial segment of superior frontal gyrus (MSFG) and in the right cerebellum VIIb/Crus II/Crus II, as well as decreases in rGMV (in blue): in the left and right middle frontal gyrus (MFG) adjacent to the opercular part of the inferior frontal gyrus (OpIFG), and in the left and right superior frontal gyrus (SFG), in ultrasound verum, relative to placebo.

Complete structural pre- and post-test data was available for *n* = 20 participants in verum, and *n* = 14 participants in placebo. We found significant clusters for both directions (increase and decrease model for ultrasound verum, clusters exceeding a threshold of *k* > 97). All significant clusters are depicted at their peak intensity coordinate in Fig. 1. Concerning increases from pre-to-post in the ultrasound verum condition, two clusters were identified: in the left anterior cingulum (ACC: − 9, 30, 39; *t* = 6.09, *k* = 320), and right cerebellum (region VIIb/Crus II; 41, − 57, − 45; *t* = 4.43, *k* = 651).

Decreases were identified in four clusters: bilateral middle frontal gyrus, including parts of the operculum of inferior frontal gyrus (right MFG/OpIFG: 30, 21, 21; *t* = 6.28, *k* = 858; left MFG/OpIFG: − 33, 24, 24; *t* = 6.07, *k* = 556), and bilateral superior frontal gyrus (left SFG: − 17, − 11, 78; *t* = 4.30, *k* = 340; right SFG: 21, 17, 42; *t* = 4.30, *k* = 256).

#### Increases and decreases in ultrasound verum relative to placebo in regional white matter volume

There were no significant changes in rWMV in the verum condition relative to placebo exceeding the cluster extent threshold (*k* > 130).

#### Changes within ultrasound placebo

There were no absolute decreases in rGMV within the placebo group, but there were two clusters of increase identified: one cluster roughly corresponding to the right entorhinal area (right Ent; *t* = 4.56, *k* = 192; 12, 2, − 41) and another cluster including parts of the left ventral diencephalon/thalamus (left ventral DC/thalamus proper; *t* = 4.44, k = 127; − 17, − 14, 5). For a figure, please see Supplementary Appendix III.

### Correlations between behavioral and regional grey matter volume changes

Change scores of rGMV (post minus pre) within the six identified clusters were correlated with change scores only of variables that exhibited a significant change in verum in the behavioral analyses. This only applied to the speed-accuracy trade-off index, which significantly declined in verum. No significant brain-behavior-correlations were identified between the change in speed-accuracy trade-off and any of the clusters (all *p* > 0.05).

For US placebo, again change scores of rGMV (post minus pre) within the two identified ROIs were correlated with change scores of variables that exhibited a significant change in placebo: somatization and the phasic arousal index. There were no significant associations, except for a trend-level, medium-sized correlation between changes within left ventral DC/thalamus proper and somatization (*r* = − 0.487, *p* = 0.078); although the data was heterogeneous, there was a tendency of smaller changes in this cluster being associated with higher levels of somatization at post-test. A plot of this association can be found in Supplementary Appendix IV.

## Discussion

The present study was dedicated to the question of whether airborne ultrasound above the human hearing range (20 kHz) affects human behavior and brain structure, which is particularly of interest given ultrasonic waves now being omnipresent in manmade environments. We reported here on the first ever conducted randomized-controlled experimental, longitudinal trial that involved an assessment of both behavioral and neuroimaging data. Key findings are summarized in the following paragraphs.

### Effects of ultrasound on human behavior

Our results suggest that there is no consistent evidence for alterations through ultrasound in any of the assessed behavioral domains (i.e., sound sensitivity, personality, self-reported psychiatric symptoms, quality of sleep, cognitive performance). There were isolated effects, with increases in self-reported symptoms in the sham condition, possibly hinting towards a nocebo effect. The latter notion adds up to a recent study that identified somatic nocebo-responses in sham-exposure to ultrasound^7^. In addition, there were isolated effects on cognitive performance variables: the phasic arousal index improved in placebo, but remained unchanged in verum, and there was a shift towards a speed strategy (vs. accuracy) in verum. This extends the existing literature insofar, as it goes beyond the evidence of somatic effects evoked by ultrasound (e.g., nausea, headache, fatigue, tinnitus^1,4^), by suggesting that ultrasound may have an effect on cognitive flexibility (decreased accuracy). However, in sum the data is inconclusive, and needs be interpreted with caution, as effects are not statistically significant after alpha-level-correction. Further experimental replication is needed.

### Effects of ultrasound on brain structure and correlations with behavior

Exploratory structural grey matter analyses (Voxel-Based Morphometry) revealed several substantial clusters that exhibited both in- and decreases in regional grey matter volume pre-to-post US exposure. Areas of relative increases included the left anterior cingulum (ACC, BA24), and right cerebellum VIIb/Crus II. In VBM-based studies, the dorsal region of ACC has been found to show diminished levels of rGMV in individuals with trauma experience^26^. Functionally, the involvement of the ACC in stress regulation has also been confirmed in several studies^27^. Speculatively, the increase in that area could indicate an increase in stress regulation in response to ultrasound exposure, but this needs further study. Area VIIb/Crus II of the cerebellum has recently been shown to be involved in demanding cognitive tasks, such as difficult n-back working memory, but also in language-related tasks, which were found to be right-lateralized^28^.

Furthermore, there were substantial bilateral frontal clusters that exhibited decreases from pre-to-post within the verum (relative to the sham) condition, involving the following areas: bilateral middle frontal gyrus and opercular part of the inferior frontal gyrus (MFG/OpIFG [BA 46, 44]). These areas are critically involved in syllable information coding (bilateral OpIFG—BA44) and semantic tasks, as well as motor aspects of speech (left OpIFG—BA44) and sustained attention as well as self-control/ inhibition (bilateral MFG—BA46). Left [BA 4, 6] and right SFG [BA 6, 8] are known to be particularly related to motor control, but also down-regulation of arousal (right SFG), as shown by a lesion-based study^29^. However, in the present study we were unable to relate these structural changes to behavioral changes that were observed in verum (i.e., speed-accuracy trade-off decrease).

Concerning white matter, no significant changes in verum relative to placebo could be identified. Given the overall pattern of results, this suggests that ultrasound may particularly induce grey matter decline in frontal areas. In addition ultrasound may not induce changes in anatomical connectivity, which however does not preclude other types of connectivity changes (i.e., functional or effective). This should be followed up in resting state and functional brain analyses.

### Effects within the Sham condition (signs of nocebo effects)

Of note, exploratory correlation analyses of identified changes within the sham group revealed that changes in somatization (mainly increases) were associated with stronger increases in a cluster including parts of the left ventral diencephalon/thalamus proper. These regions are involved in somatosensory processing and have been shown among other regions to be associated with the unconscious conditioned nocebo response^30^. However, caution is warranted in interpreting the effects within the sham group, since a control group (such as waitlist control) was missing. Therefore, the rGMV change analyses are prone to reflect changes due to changes in the MRI hardware over time and other confounders. Nevertheless, the identified effects in sham could hypothetically be attributed to maintained vigilance in expecting detecting a signal, but in the absence of any sensory input from the source, while using bodily states as a means to detect any indications of adverse effects through the source. In the verum group, there was feedback (although very likely only unconsciously processed) and there was a distinct pattern of neurological change, which is why bodily reactions might not have been in the focus of attention.

### Limitations

Our study sample was young and healthy, and not selected to be particularly sensitive to ultrasound. Follow-up investigations will need to recruit a broader, more balanced age-range and include individuals with self-reported high-sensitivity to systematically control for different possible moderators. In addition, expectations of effects need to be addressed more thoroughly, as there is evidence for nocebo responses from a prior study^7^. Of interest, nocebo responses as observed in our ultrasound condition can be prevented by psycho-education^31^, hence assessing expectation bias and a waitlist (natural) control group would be mandatory for future works. Furthermore, as the sample size of the control group was small, robustness of findings are not entirely assured especially for this group, and generally warrant replication. Nevertheless, the present study significantly contributes to narrowing down target variables, methods, and design requirements for future trials by having applied a manualized and controlled approach.

### Conclusion

Ultrasound exposure is associated with decreases in regional grey matter volume in brain areas involved in executive functioning, attention, stress regulation and inhibition (bilateral MFG/OpIFG, bilateral SFG). Increases were observed in areas that encode complex motor and working memory functions (bilateral cerebellum VIIb/Crus II), and stress regulation (left ACC). However, no brain-behavior correlations concerning ultrasound exposure could be established in this study, which is why the consequences of the identified brain alterations are speculative.

The isolated significant effects identified in our study—increases in somatization related to ultrasound-sham exposure (possible nocebo effect), increase of phasic alertness in sham (possible indication of heightened awareness due to expectation but lack of sensory input from the sham source) and cognitive flexibility with a shift towards a speed strategy (vs. accuracy) in verum (possible indication of increased hastiness as a result of ultrasound exposure)—need to be replicated.

We suggest conducting further ultrasound exposure trials and recommend including additional tasks in order to establish brain-behavior correlates, including complex motor control and coordination, demanding n-back working memory, stress regulation, speech processing, and impulse control/ inhibition (e.g., stop-signal) tasks.

## Supplementary Information

Supplementary Information.

## Data Availability

We hereby declare that our data, code and syntaxes (including a documentation of all analyses that were undertaken) are available upon request.
