# Supplementary material for: A longitudinal, randomized experimental pilot study to investigate the effects of airborne ultrasound on human mental health, cognition, and brain structure
Source: Sci Rep. 2021 Mar 12;11:5814. doi: 10.1038/s41598-021-83527-z (PMC7955070; doi:10.1038/s41598-021-83527-z)
Supplement: Supplementary file 1 — Supplementary Information. [file 41598_2021_83527_MOESM1_ESM.pdf]

A Longitudinal, Randomized Experimental Pilot Study to Investigate the Effects of Airborne Ultrasound on Human Mental Health, Cognition, and Brain Structure

APPENDIX

Ascone, L.[1], Kling, C.[3], Wieczorek, J.[3], Koch, C.[3], Kühn, S.[1,2]

[1] University Medical Center Hamburg-Eppendorf, Department of Psychiatry and Psychotherapy, Neuronal Plasticity Working Group, Martinistr. 52, 20246, Hamburg, Germany.

[2] Max Planck Institute for Human Development, Lise Meitner Group for Environmental Neuroscience, Lentzeallee 94, 14195 Berlin, Germany

[3] Physikalisch-Technische Bundesanstalt Braunschweig, Bundesallee 100, 38116 Braunschweig, Germany

Contact information of corresponding author: l.ascone-michelis@uke.de

Appendix I

1a) Design, technical details, and initial adjustment of ultrasound sources

The ultrasound sources were constructed using commercially available marten repellents. Three functional sources for the verum group and three dummy sources for the placebo group were built with identical appearance and outer behavior. The sources were designed to emit a Z-weighted sound pressure level not exceeding 90 dB SPL at typical head positions of sleeping participants. For the construction, five different commercial ultrasound sources were tested in the laboratory. Three of them were not suitable because of inappropriate signal generation or parallel emission of audible sound components, and one was excluded because of unreliable operation. Only one source, SecoRüt MS4, was suited for the purpose of the study.

The original source was designed to sweep the signal in frequency and level over time for maximum unpleasantness to successfully repel animals. Since for the study a steady signal was desired, the signal generation electronics were modified so that a stable sinusoidal signal of about 22.4 kHz was emitted at a constant level (see Appendix Figure 2). For technical reasons harmonic components at multiples of the fundamental frequency could not be avoided. Due to their high frequencies and low sound pressure levels (e.g. the first harmonic was more than 20 dB below the fundamental), these signal components were not expected to influence the experiment. The source was built in a small case of about 10 cm (length) x 7 cm (width) x 7 cm (height) which could be placed even on a small bedside table (see Appendix Figure 1). The speaker was embedded on the top side of the case sounding to a small exponential reflector mounted on top to distribute the ultrasound radially around the source. The source could easily be switched on and off. A LED light gave feedback about the power status. The source was powered by a small external mains supply. The verum sources and the placebo sources were identical setups with the only difference that the electronics in the placebo sources were decommissioned while the LED was still working. Due to the same outward appearance and

behavior, it was not possible for the participants to distinguish between placebo and verum sources.

Neither background nor exposure levels were taken at the exposure sites. In difference to the accompanying infrasound study, no measurements of the SPL in the bedrooms of the participants were performed. From related measurements in the laboratory and at workplaces (1,2) it was known that reliable values could not be expected without a sophisticated measurement procedure and including the acoustical influence of the participant's head. For practical reasons, this could not be realized in an alien bedroom. Alternatively, a comprehensive laboratory characterization of the ultrasound source was performed, adjusting them to ensure a defined maximum exposure level. Therefore, three different typical arrangements of bedroom furniture combined with three different potential head positions were simulated in the laboratory in a free field environment. Wooden boards and absorbing foams were used to simulate the acoustical behavior of surrounding walls and furniture including a single and a double bed and a nightstand. Appendix Figure 3 gives an example with a specific arrangement. Measurements in a more realistic arrangement equipped with pillows and blanket yielded comparable results. The measured exposure levels varied over a range of about 21 dB, mainly depending on the distance between the head and the source and on the arrangement, i.e. occurrence of hard-reflective walls. The observed variations of the exposure levels were comparable to measurements at other ultrasound exposure sites (1,2). Following these simulation results, the ultrasound sources were adjusted to ensure a Z-weighted sound pressure level (ref. 20 µPa) of a maximum of 90 dB for any of the tested head positions. From these findings, arrangement instructions were drawn for an appropriate positioning and handling of the sources by the setup team and participants. Finally, all assembled ultrasound sources were tested in the laboratory for long-term functionality for at least up to 24 hours. All sources showed an unavoidable warm-up time frame of about 10 minutes to 30 minutes within that the

SPL varied to about ±3 dB before reaching a plateau where the measured SPL drift was less than about 1 dB within 4 hours. Sound emission never exceeded the designed and desired level.

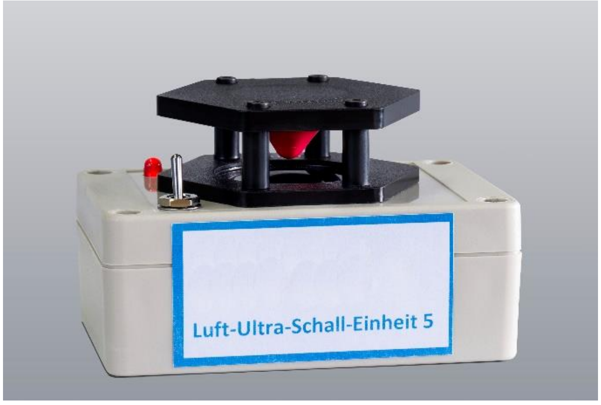

Appendix Figure 1. Photograph of one of the ultrasound sources.

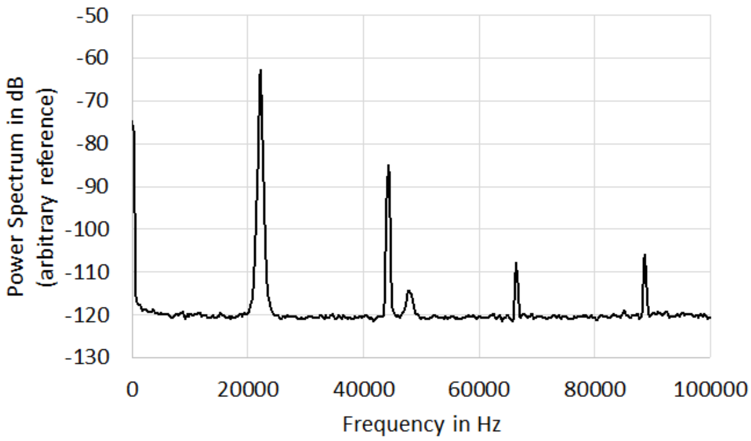

Appendix Figure 2. Exemplary power spectrum of one of the ultrasound sources, the fundamental frequency at about 22.4 kHz and harmonic components can be identified.

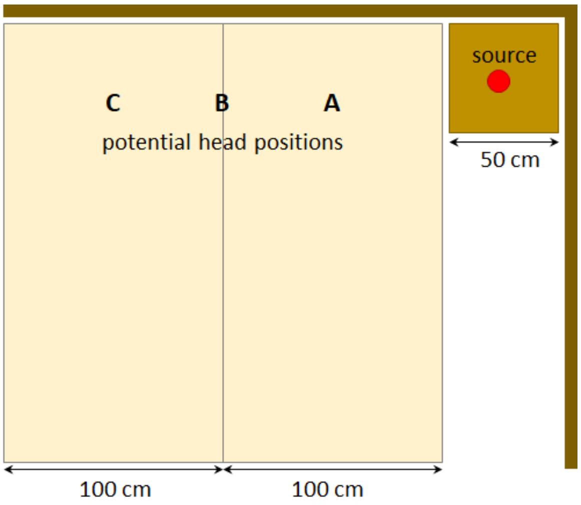

Appendix Figure 3. Bedroom simulation arrangement 1 with two reflective walls, a nightstand and a double bed. The source is located at the nightstand. The sound pressure levels at three potential head positions were measured to about (A) 81 dB, (B) 79 dB and (C) 74 dB (maximum Z-weighted sound pressure levels in reference to 20 µPa).

1b) Description of standardized on-site procedure and assessments

All measurements on-site (in the participants' bedrooms) were taken after 6 pm (at 6:40 pm on average; range between 6:00 and 10:15 pm), with two exceptions due to a shift work situation (measurements taken at 1:30 and 3:00 pm). In order to realize the on-site assessments in a standardized manner, a measurement protocol was developed, which will be briefly described in chronological order in the following paragraphs.

(1) First, it was explained to all participants that we would install and calibrate the sound source in their bedroom, which would require them to leave the room later in the process. Next, they were interviewed concerning their typical sleep interval and about potential additional noise sources in the night. Their preferred and most frequent sleep constellation was assessed (i.e. position of doors and windows). We communicated to them that an 'all-closed' constellation would be preferable for reasons of controllability of the experiment, however, if participants communicated that they clearly preferred other constellations and would consistently use them for the duration of 28 days, this was tolerated. The participants were now sent out of the room, asking them to close the door, while explaining to them that absolute quiet was needed for the measurement procedure.

(2) We now measured the room (height, width, length) and did a quick sketch, that also included noting larger furniture or any particularities within the bedroom. The location of the sound source, including its distance from the bed (head-side) and height (the loudspeakers were height-adjusted to the location of the head). Pictures of all bedrooms were also taken from four different angles. Notes about any peculiarities in the room were taken.

(3) The source was switched on and after a warm-up phase, the experimenter tested its audibility at the bedside. The distance of the source from the head-side on the bed was measured. The height of the loudspeaker stand was adjusted so that the source was at the same height as the head.

(4) The sound sources were programmed using a time switch to emit sound for 8 hours, covering the participants’ reported habitual bedtime (sleep) interval, including programming potential variations.

(5) Prompters to remind participants of the room constellation were placed in the bedroom. In addition, they received a sleep protocol for assuring compliance, checking the constellation every evening before going to bed and documentation of bedtime, sleep time, wake-up-time, and any deviations.

1c) On-site constellation details and exposure levels

Neither background nor exposure level measures were taken in-situ. The average distance from the site of the head (bed) at which the ultrasound verum sources (sample size n = 21) were placed was 1.64 m (SD = 0.69 m). The average distance in the ultrasound placebo group was M = 1.66 m (SD = 0.76m), which did not differ significantly from that of the verum group ($t(27) = -0.06$; $p = .955$). The estimated average room volume (based on multiplication of measures of room length, width and height) was 37.43 m^3 (SD = 7.29 m^3) in the ultrasound verum group.

Appendix II: Cognitive Test Parameters

Concerning *alertness*, tonic and phasic alertness can be differentiated. Tonic arousal is a good proxy for wakefulness, where the participant is in a state of willfully maintaining his or her attention and accordingly responds quickly to an upcoming event. In the TAP this is implemented as a reaction time (RT) task, where an 'X' is centrally presented on the screen, which the participant is asked to respond to with a button press as fast as possible. RTs are measured under two conditions - with and without a preceding warning tone – in four blocks (total duration: 4.5 minutes). In the present study, the median RTs for trials with vs. without warning signal were used, as recommended, as indicators for tonic and phasic alertness. In addition, the program provides an index of phasic alertness, which contrasts test performance in trials with vs. without warn tone, hence providing an estimate of 'benefit' from the acoustic prompter. In addition, total anticipations are recommended as a measure for disinhibition.

Sustained attention refers to the capacity to concentrate on a routine task for a longer time period (15 minutes in the case of the test). In the TAP, a successive discrimination task is implemented. Participants are instructed to press a button when two stimuli with the same form (e.g., an oval) appear after one another. Out of 450 stimuli, only 54 are targets, and these are evenly distributed across the task, within three 5-minute-intervals. Hence, participants need to sustain their concentration in order not to miss targets. We used the number of omissions as indicator for potential changes in sustained attention.

Flexibility is the ability to redirect attention from one to another object, modality, or task. It is a heterogeneous construct, ranging from willfully shifting the sensory focus to strategic control of complex coordinated behavior. In the TAP, a letter and a number are presented left and right (or reversely) simultaneously on the screen. The participant has the instruction to shift to which stimulus type he or she must respond for each trial, starting responding to the side (left or right button) where the letter is presented, then shifting to responding to the side where the number is presented, and so on, for 100 trials with a duration

of about 3 minutes. Most important parameters are the total performance index and the speed-accuracy (trade-off) index. The total performance index integrates both the correctness and speed of reactions, where positive values indicate above-average fast and correct reactions. Negative values accordingly indicate below-average performance (high error rate, slow reactions). If the performance is (approximately) zero, this indicates average total performance. A negative speed-accuracy index denotes a relatively high error rate and fast responses (speed strategy), whereas positive values indicate low error rates but long RTs (accuracy strategy).

Divided attention refers to the ability to simultaneously attend to concurrent tasks. In the TAP this is operationalized in a task where participants are simultaneously presented with stimuli of two different modalities: vision and hearing. While the participant must discriminate between, and respond to, visual target stimuli, at the same time successive high and low beep tones are presented. Responses are required to either when a visual target stimulus (20 out of 175 stimuli) or two successive high or low tones (20 pairs out of 287 tones) are presented. The total task takes about 3.5 minutes. The number of omitted targets is the most important test parameter, as indicating the inability to attend to both modalities simultaneously.

Incompatibility effects occur in conflict situations, where divergent information generates a conflict in reaction tendencies. The TAP applies the Simon-paradigm. The aim of the task is to respond with the correct hand, as indicated by the direction that is pointed out by an arrow. The arrow however not only points into the direction of the hand with which participants have to react, it also appears on the right vs. left side of the screen, creating compatible (e.g., pointing left, appearing on the left side) vs. incompatible (e.g. pointing left, appearing on the right side) information. Incompatible information results in conflicting reaction tendencies, whereby the irrelevant, incompatible information needs to be discarded. Incompatibility is manifest in slower RTs, while compatibility leads to faster RTs. In total, there are 60 trials, of which 15 are compatible and 15 incompatible on each side (left, right). Of interest are the parameters: errors in incompatible conditions and validity x side interactions.

The latter quantifies the incompatibility effect, with lower values denoting a stronger and higher values a weaker effect.

Covert shift of attention describes the ability to shift the attentional focus without making eye movements (i.e., endogenous attentional shift). A target stimulus appears at the left or right side of the screen, whereby its appearance is preceded by a cue arrow-stimulus, presented centrally on the screen, which points to the congruent (where the stimulus is truly going to appear) or incongruent side (opposed to where the stimulus is going to appear). The task includes 100 trials, of which 20 are invalid and 80 are valid cued trials. Particularly meaningful for interpreting the task performance is the validity x side index, which indicates the shift of attention ability. Lower values indicate more pronounced problems to willfully re-orient attention after an invalid cue while higher values indicate better performance.

The *GoNogo* task measures the inhibition capacity of the respondent. A primary, externally triggered impulsive reaction tendency needs to be suppressed in favor of an internally controlled reaction. In the TAP this is implemented by presenting a successive sequence of 5 different types of stimuli, out of which 2 types are targets. All stimuli look relatively similar, which is why oftentimes responses need to be suppressed. It is recommended to evaluate the number errors as a marker of lower inhibition.

Appendix III

Descriptive pre-post data (means and standard deviations) for all behavioral variables

Variable	Ultrasound verum – T1 (n=21)	Ultrasound verum – T2 (n=21)	Ultrasound placebo – T1 (n=14)	Ultrasound placebo – T2 (n=14)
Sensitivity				
High frequency sensitivity	18.8 (12.6)	16.8 (11.7)	17.4 (7.57)	16.6 (9.28)
Normal sound sensitivity	45.5 (12.4)	45.0 (11.8)	45.2 (13.8)	43.9 (14.5)
Symptoms and Sleep				
BSI total	16.1 (14.3)	16.1 (19.3)	16.4 (13.4)	22.5 (18.9)
BSI somatization	1.10 (1.50)	1.00 (1.34)	0.71 (1.38)	1.57 (1.70)
BSI depressive symptoms	1.80 (2.09)	1.90 (3.28)	1.64 (1.91)	2.50 (2.93)
BSI anxiety symptoms	2.40 (2.54)	2.00 (2.41)	1.93 (2.62)	2.50 (2.90)
ESS sleepiness	8.86 (4.90)	8.00 (4.67)	9.93 (4.23)	9.86 (4.69)
PSQI – sleep quality (total)	5.70 (2.05)	5.74 (2.56)	5.71 (1.68)	5.85 (1.72)
PSS perceived stress	23.9 (7.25)	23.3 (7.27)	22.0 (3.96)	25.3 (7.45)
Alertness				
Median RTs tonic arousal	240.4 (33.9)	236.1 (32.0)	236.3 (28.6)	243.2 (36.5)
Median RTs phasic arousal	239.2 (27.1)	231.9 (30.3)	239.6 (31.5)	226.2 (23.5)
Phasic alertness index	.003 (.070)	.017 (.060)	-.013 (.069)	.068 (.095)
Anticipations after warn tone	1.10 (1.55)	1.00 (1.00)	1.36 (2.17)	0.79 (0.97)
Sustained Attention (WM)				
Omissions (min. 10-15)	4.10 (3.89)	4.48 (5.94)	5.14 (4.13)	6.93 (7.04)
Flexibility				
Speed-accuracy index	0.81 (7.00)	-3.06 (9.69)	0.21 (8.09)	3.16 (9.60)
Total performance index	3.84 (8.24)	6.50 (8.37)	-1.30 (4.57)	3.97 (6.45)
Divided Attention				
Omissions (total)	2.14 (4.60)	2.86 (5.01)	2.50 (2.68)	3.64 (3.48)
Incompatibility				
Incompatibility effect	4.95 (8.50)	5.65 (8.96)	4.56 (5.50)	6.92 (7.80)
Errors incompatible	1.71 (1.90)	1.33 (1.43)	1.50 (1.51)	1.29 (1.27)
Covert Shift of Attention				
Validity x side index	2.00 (2.09)	1.77 (3.01)	1.43 (1.65)	0.83 (0.92)
GoNoGo				
Errors (total)	0.30 (0.47)	0.30 (0.47)	0.79 (1.12)	0.71 (0.73)
Personality				
Openness	13.5 (4.41)	13.8 (4.81)	15.0 (4.72)	13.6 (5.36)
Neuroticism	6.75 (4.53)	7.14 (4.55)	5.86 (4.85)	7.79 (5.44)
Conscientiousness	17.8 (4.06)	18.3 (4.12)	18.1 (3.25)	17.9 (4.08)
Extraversion	14.8 (2.63)	14.4 (3.31)	14.3 (3.63)	14.8 (4.10)
Agreeableness	6.85 (3.33)	7.57 (3.10)	5.64 (3.27)	6.50 (4.00)

Appendix IV

Structural findings of rGMV changes and correlations with behavioral variables within the ultrasound placebo group

For details on the analysis procedure, please refer to the main paper. There were no absolute decreases in rGMV within the placebo group, but there were three clusters of increase identified: one cluster roughly corresponding to the right entorhinal area (right Ent; t = 4.56, k = 192; 12, 2, -41) and another including parts of the left ventral diencephalon/ thalamus (left ventral DC/ thalamus proper; t = 4.44, k = 127; -17, -14, 5); see Figure 4 for details. Figure 5 shows the identified trend-level significant correlation between the structural changes in the left ventral DC/ thalamus proper cluster with changes in somatization. No further significant associations were identified.

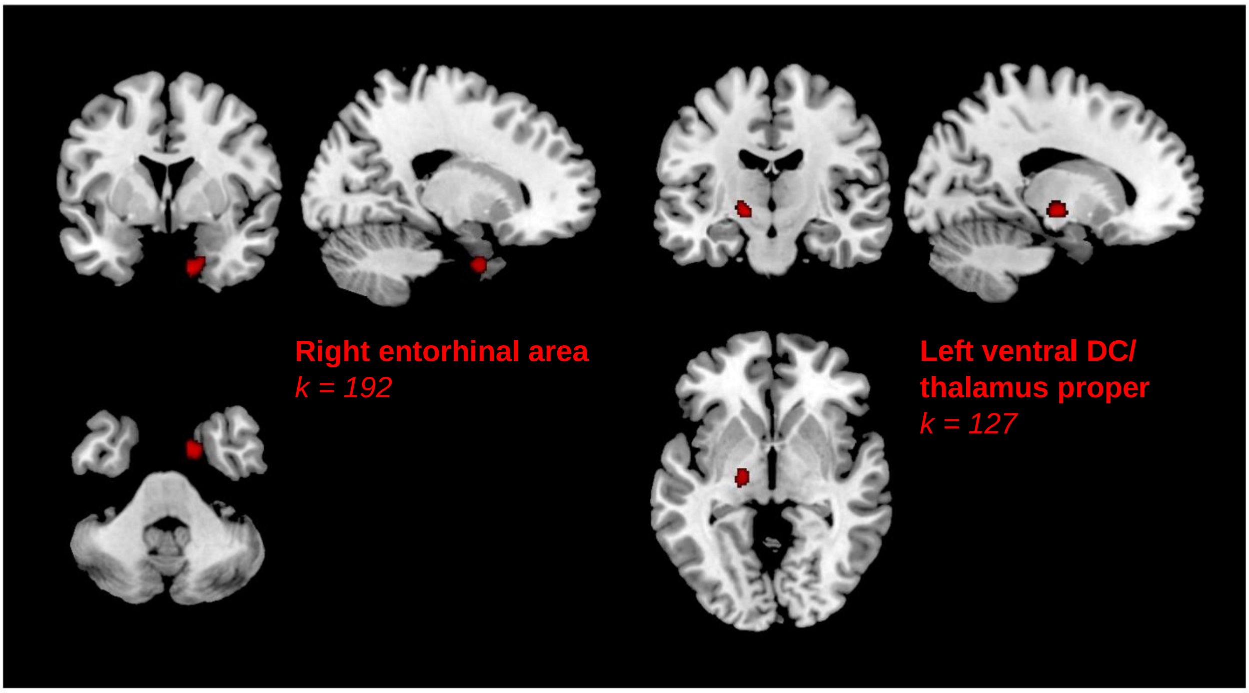

Appendix Figure 4. Graphical depiction of identified significant clusters in the VBM analysis of increases in rGMV within the ultrasound placebo condition.

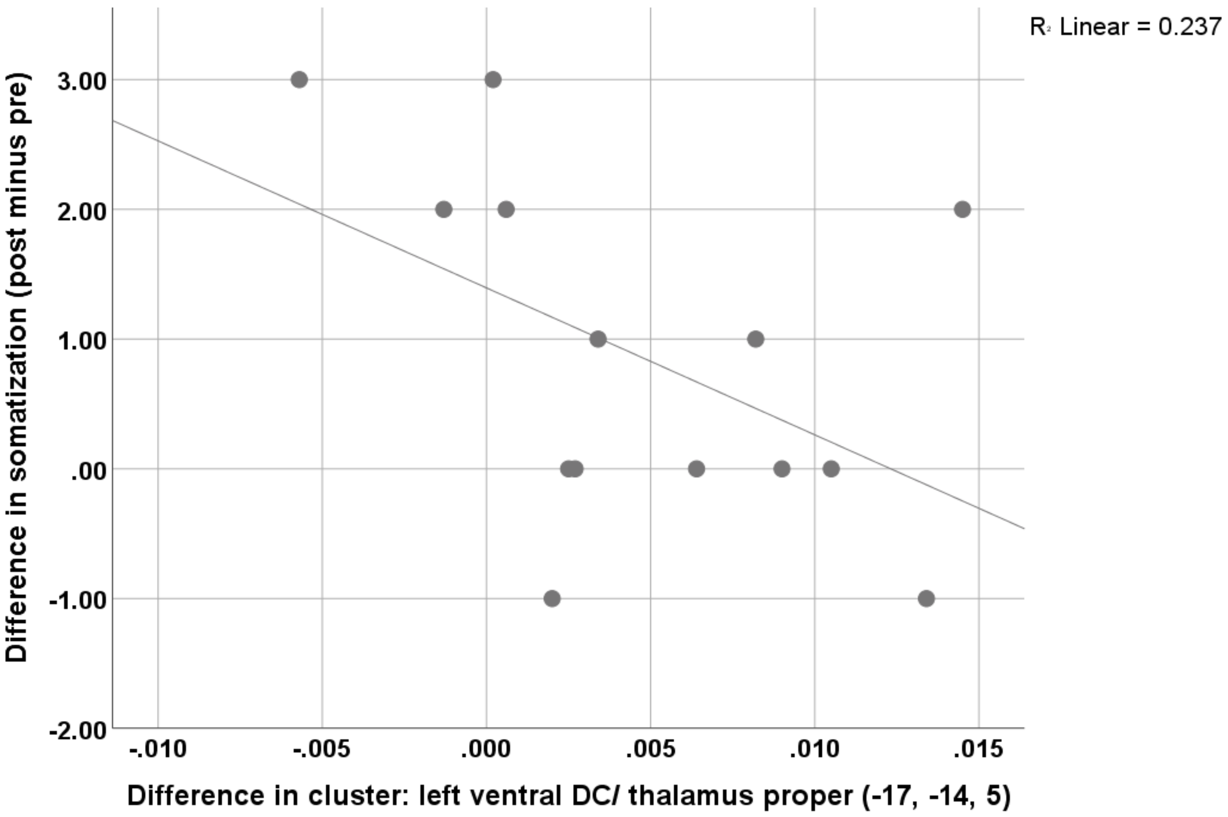

Appendix Figure 5. Correlation scatter plot with linear fit line for the association between differences at pre and post-test within the cluster left ventral DC/ thalamus proper (mean increase of rGMV in placebo) and somatization (*r* = -.487, *p* = .078).
